# Mechanical Grinding Preparation and Characterization of TiO_2_-Coated Wollastonite Composite Pigments

**DOI:** 10.3390/ma11040593

**Published:** 2018-04-12

**Authors:** Wanting Chen, Yu Liang, Xifeng Hou, Jing Zhang, Hao Ding, Sijia Sun, Hu Cao

**Affiliations:** 1 Beijing Key Laboratory of Materials Utilization of Nonmetallic Minerals and Solid Wastes, National Laboratory of Mineral Materials, School of Materials Science and Technology, China University of Geosciences (Beijing), Beijing 100083, China; wantingchen123@163.com (W.C.); ssjcugb@163.com (S.S.); caohu2001@163.com (H.C.); 2 School of Materials Science and Technology, Shenyang University of Chemical Technology, Shenyang 110142, China; liangyuaadd@126.com; 3 Dalian Huanqiu Minerals (Group) Corp., Building 3F, No. 159 Jinma Road, Dalian Economic and Technological Development Zone, Dalian 116600, China; dljoycezhang@aliyun.com

**Keywords:** wollastonite, TiO_2_, composite particles, mechano-chemical method, pigment properties

## Abstract

TiO_2_-coated wollastonite composite pigments were prepared by the mechano-chemical grinding of wollastonite and TiO_2_ powder together in a wet ultrafine stirred mill. X-ray diffraction, scanning electron microscopy, X-ray photoelectron spectroscopy and infrared spectra were used to investigate the microstructures and morphologies of the composite and the reaction mechanism. The results indicate that the TiO_2_-coated wollastonite composite pigments have similar properties to titanium dioxide pigment, showing much better properties than dry and wet mixing of wollastonite and TiO_2_. The hiding power of TiO_2_-coated wollastonite composite pigments (45% TiO_2_) is 17.97 g/m^2^, reaching 81.08% of titanium dioxide. A firm combination between wollastonite and TiO_2_ is obtained through a surface dehydroxylation reaction during the mechano-chemical method.

## 1. Introduction

Titanium dioxide pigment is a kind of functional powder material made of crystal phase titanium dioxide particles at a scale of 200–300 nm. Titanium dioxide has high hiding power, high achromatic force, high gloss and whiteness because of its high refractive index (the refractive index of rutile is 2.7 and the refractive index of anatase is 2.3). It also shows excellent weather resistance and good dispersity, as well as photocatalysis properties [1,2,3,4,5]. All of these make titanium dioxide the most widely used white pigment in many areas such as coating, plastic, decorative paper, ink and color rubber products. However, there are many problems in terms of resourcing, the environment and the cost with the process of titanium dioxide production and application. Titanium dioxide particles aggregate and have poor compatibility with an organic application system, which also limits the efficiency of using titanium dioxide and increases the existing pressure on its production and application [6,7]. In order to improve the dispersity and compatibility of the TiO_2_ particles with the matrix, as well as to maximize its pigment function, a new kind of inorganic pigment that can coat TiO_2_ firmly onto the surface of inorganic particles, such as nonmetal minerals, is widely considered. Until now, some TiO_2_ composite pigments were prepared by TiOSO_4_ hydrolysis and calcination on the surfaces of barite [8], kaolinite [9,10,11,12] and mica [13,14,15] and some other TiO_2_ composite pigments were prepared by way of a mechano-chemical method using barite [16], calcium carbonate [17,18], brucite [19], calcined kaolin [20], sericite barite [21] and silica [22] as the core and TiO_2_ particles as the shell.

Wollastonite, a natural and abundant mineral, has excellent physical and chemical properties such as low oil absorption, high chemical stability, high temperature resistance and easy processing, and has widely been used in the plastic, rubber, paint and ceramic industries. It has a higher refractive index (1.63), low oil absorption capacity and active groups such as -OH on the surface, which are beneficial for making composite pigments together with TiO_2_. Compared with titanium dioxide, the preparation and application of TiO_2_-coated wollastonite composite pigments can reduce the cost of products and increase the additional value of wollastonite. Zhao [23] and Yang Shaofeng [24] produced TiO_2_-coated wollastonite composite pigments by way of the chemical precipitation method. Their results showed that the particle size and the pretreatment of wollastonite in titanium sulfate solution were key factors to the success of the experiments. However, a large amount of acid waste water and solid waste such as titanium gypsum were produced using this method, causing serious environmental pollution. Therefore, in this paper, TiO_2_-coated wollastonite composite pigments were prepared using a mechanical grinding method and their pigment properties and microstructure were characterized. Finally, the interreaction and the composite mechanism between wollastonite and TiO_2_ in the water system from the perspective of interfacial bonding were analyzed.

## 2. Materials and Methods 

### 2.1. Raw Materials and Reagents

Wollastonite raw material, with the chemical formula Ca_3_(Si_3_O_9_), was produced in Jilin province, China. Its white degree is 94%, hiding power 272.65 g/m^2^, oil absorption 11.2 g/100 g, d_50_ 15.25 μm, and d_90_ 58.65 μm. The TiO_2_ used in this study is the commercial product of anatase titanium dioxide pigment produced by Henan Baililian chemical industry Co. Ltd., Henan, China. Its white degree is 96.2%, d_50_ 0.74 μm, d_90_ 18.50 μm, hiding power 14.57 g/m^2^, and oil absorption 25.03 g/100 g. Chemical linseed oil was also used in the experiments. 

### 2.2. Preparation Method

**Figure 1:**
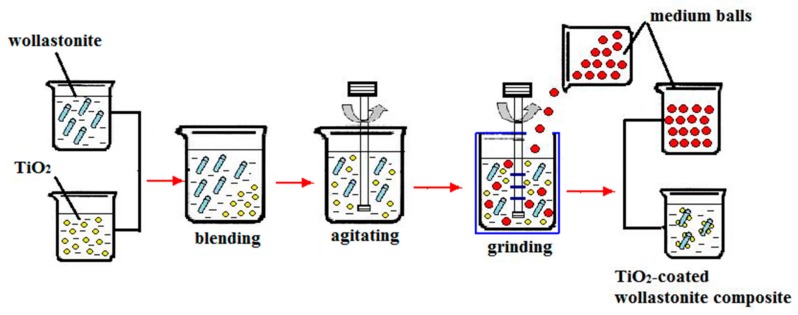
The flow chart for the preparation of TiO_2_-coated wollastonite composite.

The process of preparing TiO_2_-coated wollastonite composite pigments is shown in Figure 1. First, wollastonite was wet ground in a GSDM-S3 (Beijing gosdel power&technology Co. Ltd., Beijing, China) type superfine stirring mill and TiO_2_ powder was dispersed by mixing. Second, the wollastonite slurry and TiO_2_ slurry were mixed and ground. The purposes of blending and grinding are: (1) to ensure that the wollastonite and TiO_2_ particles can be dispersed and their surface can be activated, promoting reactions such as hydration and hydroxylation in the liquid medium; (2) to increase the collision possibility between wollastonite and TiO_2_ particles by inputting high energy during the grinding process, overcoming the repulsive interaction energy barrier, achieving a firm combination between the wollastonite and TiO_2_ particles. 

### 2.3. Characterization

The pigment properties of TiO_2_-coated wollastonite composite pigments were evaluated by testing their oil absorption and hiding power. Oil absorption can be tested by way of the China national standard GB/T5211.15-2014 [25] (the 15th part of the common testing method for pigment and extender pigment) and hiding power can be tested by way of the China vocation standard HG/T3851-2006 (the testing method for pigment hiding power).

Oil absorption was tested by putting 1–2 g of sample on the glass plate and adding linseed oil drop by drop. A special knife was used during the whole process in order to ensure the linseed oil made full contact with the sample. Three to five drops of linseed oil were added at the beginning, while at the end, the linseed oil should be added drop by drop. Once the sample and the linseed oil formed a roll, and did not collapse when the knife lifted the roll, the test was finished. The whole operation should be finished in 15 to 20 min.

Relative hiding power (*E*) refers to the ratio of hiding power of the pigment relative to that of TiO_2_, the formula is as follows:

(1) $$E=\frac{S_{CT}}{S_{T}}\times 100\%$$

where *S_CT_* and *S_T_* are the areas covered entirely by TiO_2_-coated wollastonite composite pigments and pure TiO_2_ pigment respectively, in m^2^/g. 

According to the definition of hiding power, *S_CT_* = 1/*H_CT_*, *S_T_* = 1/*H_T_*, so the value can be calculated as follows:

(2) $$E=\frac{H_{T}}{H_{CT}}\times 100\%$$

where *H_CT_* and *H_T_* refer to the hiding power of TiO_2_-coated wollastonite composite pigment and pure TiO_2_ pigment respectively, which is the least quantity of the pigments that can entirely cover the black-white lattice board in the unit area in g/m^2^. Obviously, the difference (Δ*E* = *E* − *E*_0_) between the composite pigment *E* value and the TiO_2_ compound proportion (*E*_0_, %) shows the promotion of TiO_2_ hiding power in the composite, which reflects the contribution of particle composite technology as well. 

The microstructure and morphologies were investigated by X-ray diffraction (XRD) and scanning electron microscope (SEM, S-3500N, Hitachi, Tokyo, Japan). The XRD analyses were conducted on a Rigaku D/max-rA (12 KW) X-ray powder diffractometer (XRD, D/MAX-2000, Rigaku Corporation, Tokyo, Japan) operated with Cu Kα radiation at 40 kV and 100 mA and with a scanning speed of 0.5° (2θ)/min. The mechanism of the mechano-chemical reaction between wollastonite and TiO_2_ was studied by infrared spectroscopy (IR, Equinox55, Bruker, Billerica, MA, USA) within the range of 4000–400 cm^−1^ using the standard KBr pellet technique. X-ray photoelectron spectra (XPS) were obtained using the radiation of Al Kα line (1486.6 eV, 300 W) as the excitation source. Binding energies were referenced to the C1s peak at 284.8 eV. The pigment properties were evaluated in terms of their hiding power (according to the National Industry Standard HG/T3851-2006), oil absorption value (according to the National Standard GB/T5211.15-2014) and whiteness (Digital Whiteness Instrument, SBNY-1, Shanghai Yuefeng Instrument& Meters Co. Ltd., Shanghai, China).

## 3. Results and Discussion

### 3.1. The Property and Microstructure of TiO_2_-Coated Wollastonite Composite Pigments 

#### 3.1.1. The Pigment Properties and Comparison of TiO_2_-Coated Wollastonite Composite Pigments

Table 1 presents the properties of TiO_2_-coated wollastonite composite pigments (the ratio of TiO_2_ was 45%), such as oil absorption, whiteness, hiding power, relative hiding power (*E*) and lifting proportion (Δ*E*). The properties of wollastonite, TiO_2_ raw material and their mixtures, obtained by wet and dry agitation, are listed in Table 1 in order to make a comparison.

From Table 1, it can be seen that the pigment properties of TiO_2_-coated wollastonite composite pigments are much better than those of wollastonite. The hiding properties were enhanced (the hiding power value decreases from 272.65 to 17.97 g/m^2^). The hiding power of TiO_2_-coated wollastonite composite pigments is 17.97 g/m^2^, relative to 81.08% of anatase titanium dioxide (used TiO_2_), which is 36.08% higher than the proportion of TiO_2_, indicating that the composite pigments have similar pigment properties to titanium dioxide. 

Table 1 also shows that the hiding power of the wollastonite and TiO_2_ (same ratio of composite particles) wet mixture is 21.56 g/m^2^ (relative hiding power 67.58%), while the relative hiding power of dry mixture is only 63.24%. Therefore, it is clear that a synergistic effect has formed in the TiO_2_ coated wollastonite composite, while there is almost no synergistic effect formed in the wet mixture or the dry mixture.

#### 3.1.2. The Morphology of TiO_2_-Coated Wollastonite Composite Pigments 

**Figure 2:**
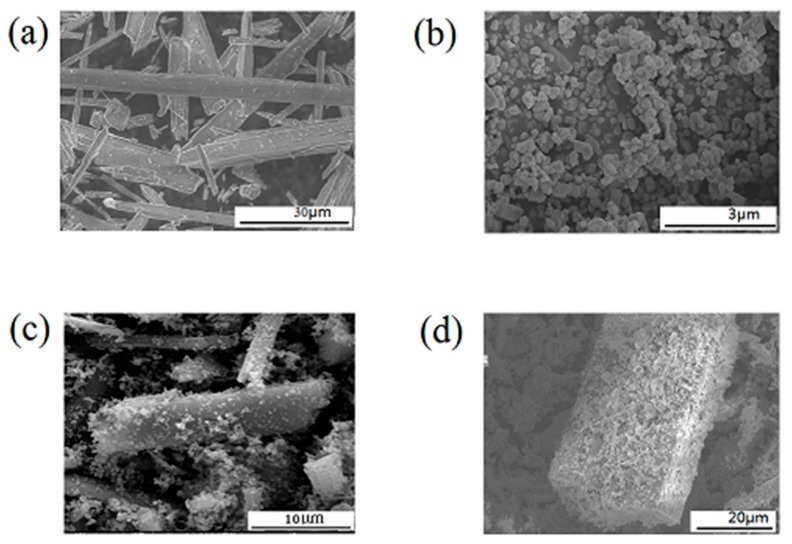
The scanning electron microscope (SEM) images of wollastonite, TiO_2_ and TiO_2_-coated wollastonite composite pigments. (**a**) Wollastonite raw material; (**b**) anatase TiO2 raw material; (**c**,**d**) TiO_2_-coated wollastonite composite pigments).

**Figure 3:**
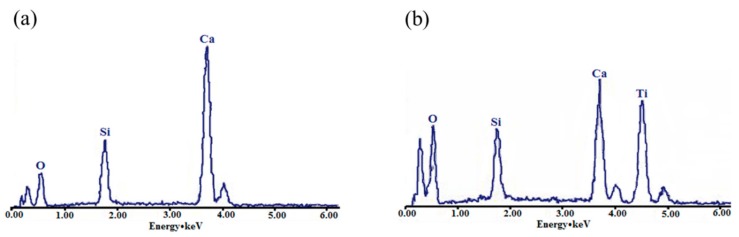
The element energy spectrum (EDS) of wollastonite and TiO_2_-coated wollastonite composite pigments (**a**) wollastonite; (**b**) TiO_2_-coated wollastonite composite pigments.

In order to observe the morphology of TiO_2_-coated wollastonite composite pigments, the scanning electron microscope (SEM) images of wollastonite, TiO_2_ and TiO_2_-coated wollastonite composite pigments were obtained and shown in Figure 2. The EDS results of TiO_2_ and the TiO_2_-coated wollastonite composite pigment are shown in Figure 3. 

Figure 2a shows that wollastonite raw material particles have a stick shape with a fair smooth surface and their length is about 10–40 μm. Figure 2b shows that TiO_2_ raw material particles have a regular cake shape with a size of about 0.3 μm, which is the best size for white pigments. From Figure 2c, we could see that the shape and outline of TiO_2_-coated wollastonite composite pigments are similar to wollastonite raw material particles, which still presents a stick shape. Figure 2d is a larger version of Figure 2c, which shows that the particle surface of TiO_2_-coated wollastonite composite pigments is no longer smooth and was covered by a large number of fine particles uniformly and compactly. Obviously, this is due to the TiO_2_ coating on the surface of wollastonite. The surface element energy spectrum analysis (EDS, Figure 3) of wollastonite before and after using the mechano-chemical method have confirmed the results. Figure 3 shows that wollastonite raw material is only composed of three elements, Si, Ca, O, which reflects the component characteristics of CaSiO_3_. A high Ti intensity spectral peak appears and the spectral peak of Ca declines in the EDS spectrum of TiO_2_-coated wollastonite composite pigments. This clearly indicates the result of a TiO_2_ covering on the surface of wollastonite. Therefore, it shows that the coating of TiO_2_ on the surface of wollastonite is uniform and orderly.

**Figure 4:**
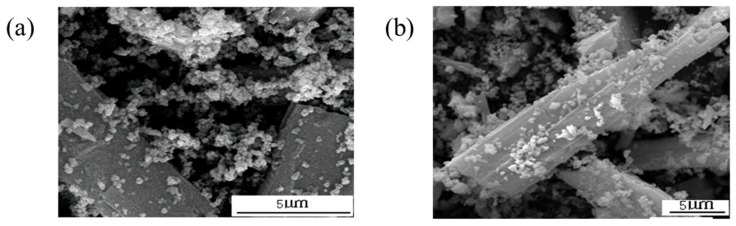
The SEM images of wollastonite and TiO_2_ mixture particles. (**a**) Dry mixtures; (**b**) Wet mixtures.

In order to further understand the differences between ordered composition and a simple mixture of the wollastonite and TiO_2_ particles and explain the importance of the particle coating, the SEM images of dry and wet mixtures of wollastonite and TiO_2_ are shown in Figure 4. Figure 4 shows that in the dry mixtures of wollastonite and TiO_2_ raw materials, the vast majority of the wollastonite surface presents a “naked” state only, with scattered TiO_2_ particles coating it, and a large number of TiO_2_ particles were in a dispersion and aggregation state and did not form a composite with the wollastonite. In wet mixtures, there were more TiO_2_ particles coated onto the surface of wollastonite than in dry mixture and there were less TiO_2_ particles alone, which indicated that the wet mixtures had a better-ordered composition than the dry method. However, its pigment properties were poorer than those of the wollastonite-TiO_2_ composite particles. Simple agitation and mixing cannot achieve the properties of an ordered coating composite pigment. 

### 3.2. The Essence of Combination Reaction between Wollastonite and TiO_2_ Particles

#### 3.2.1. Crystal Structure Analysis 

**Figure 5:**
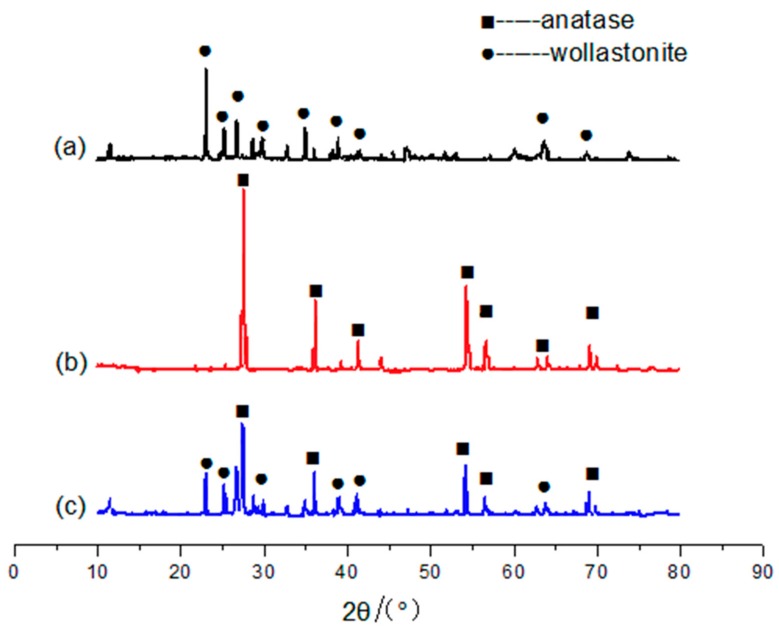
The X-Ray Diffraction (XRD) patterns of (**a**) wollastonite; (**b**) TiO_2_ (anatase type) and (**c**) TiO_2_-coated wollastonite composite pigments.

The XRD patterns of wollastonite raw material, anatase type TiO_2_ raw material and TiO_2_-coated wollastonite composite pigments are shown in Figure 5. It can be seen that both the wollastonite raw material and anatase type TiO_2_ raw material were composed of their own phase with no impurity included. After combination, only diffraction peaks of the wollastonite and TiO_2_ crystalline phase appear in the XRD pattern of TiO_2_-coated wollastonite composite pigments and no new phase occurred, which means that the composite still kept its original phase composition. These results indicate that the reaction between wollastonite and TiO_2_ only occurs on the interfaces.

#### 3.2.2. The Reaction Characteristics between Wollastonite and TiO_2_ Particles

**Figure 6:**
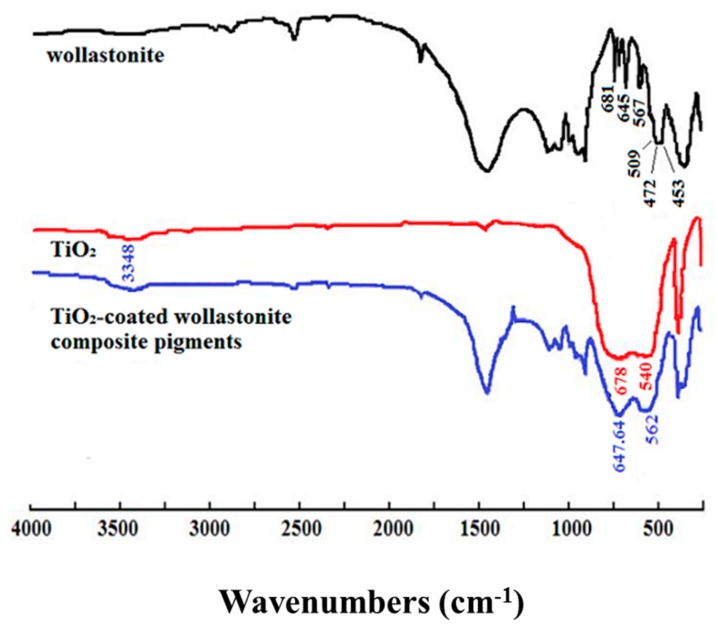
**Fourier-Transform Infrared** (FTIR) spectra of wollastonite particles, TiO_2_ particles and the composite particles.

Figure 6 shows the Fourier-Transform infrared spectra (FTIR) of wollastonite particles, TiO_2_ particles and TiO_2_-coated wollastonite composite pigments prepared by way of the mechano-chemical method. In the FTIR spectrum of wollostonite, six absorption bands (wave number 1085, 1060, 1018, 967, 927 and 902 cm^−1^), caused by Si-O-Si antisymmetric stretching vibration, O-Si-O symmetric and antisymmetric stretching vibration appear in the range of 1100–850 cm^−1^ and the two absorption bands (681 and 645 cm^−1^) caused by Si-O-Si symmetric stretching vibration appear in the range of 750–600 cm^−1^. In addition, four absorption bands (567, 509, 472 and 453 cm^−1^) caused by Si-O flexural vibration and Ca-O stretching vibration appear at low wave number. The absorption bands caused by O-H stretching and flexural vibration appear at 3438 and 1642 cm^−1^ respectively. The absorption bands at 678 and 540 cm^−1^ correspond to the Ti-O stretching vibration.

The symmetrical stretching vibration absorption from wollastonite Si-O-Si radical groups (681 and 645 cm^−1^) and stretching vibration from the Ti-O bond of TiO_2_ (678 and 540 cm^−1^) do not appear in spectra of TiO_2_-coated wollastonite composite pigments, while the new absorption bands caused by the chemical combination between Si-O-Si and Ti-O at 647.64 and 562 cm^−1^ appear. Therefore, it can be concluded that dehydroxylation has occurred on the surface of the two particles and a Si-O-Ti chemical bond was formed, which made the TiO_2_ particles coat tightly onto the surfaces of the wollastonite particles.

**Figure 7:**
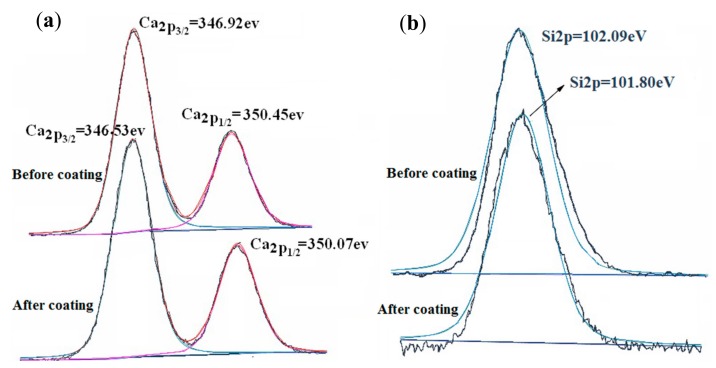
X-ray photoelectron spectra (XPS) of wollastonite before and after coated by TiO_2._ (**a**) Ca_2__p3/2_ (**b**) Si_2P_.

The XPS of wollastonite before and after coating with TiO_2_ are shown in Figure 7a,b. The binding energy of Ca_2__p3/2_ and Si_2P_ of TiO_2_-coated wollastonite composite pigments is 346.53 and 101.80 eV, respectively, after using the mechano-chemical method, while before using the mechano-chemical method the binding energy of Ca_2P3/2_ and Si_2P_ is 346.92 eV and 102.09 eV. Obviously, the chemical environment of Ca and Si has changed. Therefore, it can be concluded that the chemical reaction between wollastonite and TiO_2_ occurred on their interface.

**Figure 8:**
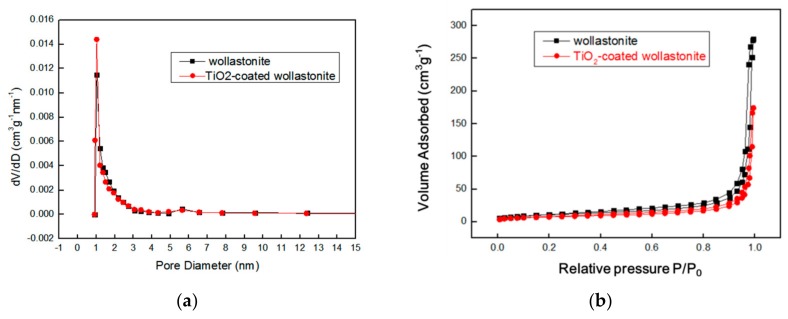
(**a**) Pore diameter distribution of wollastonite and TiO_2_-coated wollastonite (the final product); (**b**) the isotherm of N_2_ adsorption-desorption on wollastonite and TiO_2_-coated wollastonite (the final product).

N_2_ physisorption measurements were also performed on both wollastonite and TiO_2_-coated wollastonite composite (Figure 8). It can be seen that the pore diameter of wollastonite is 0.762 nm, while the pore diameter of the composite is 0.76 nm. The adsorption-desorption isotherm corresponds to the adsorption behavior of non-multiporous materials. Both wollastonite and the TiO_2_-coated wollastonite composite have almost no pores on their surfaces, which is in accordance with their structures.

### 3.3. The Composite Model between Wollastonite and TiO_2_ Particles

#### 3.3.1. The Surface Morphology of Wollastonite Particles

The surface morphology of inorganic particles (e.g., minerals) depends on factors such as element composition, crystal structure, cleavage and rupture behavior. Wollastonite is a kind of chain structure silicate with its structure in the form of single chain using three [SiO_4_] tetrahedrons as repeating units. The chain extends along the b axis and the chain gap is filled by Ca^2+^. Therefore, wollastonite has perfect cleavage characteristics along (100) in the shape of a plate, a column and a needle. Si-O and Ca-O are the main bonds in wollastonite with bond energies of 443.08 and 133.76 kJ/mol, respectively. The strong Si-O bond and the weak Ca-O bond make wollastonite break down easily along the weak Ca-O bond in the process of mechanical shock or grinding, leading Ca^2+^ and SiO_3_^2−^ exposed in an unsaturated state, with Si-O and O-Si groups appearing on the surface of wollastonite particles. Among them, the Si-O components come from the [Si_3_O_9_] single chain, with a saturated O atom located on the surface with a lone pair electrons and a Ca-O broken bond that comes from the fracture of the [CaO_6_] octahedron chain, with both Ca and O in the unsaturated state. The Si-O, O-Si and Ca-O components have the following hydrolysis phenomenon in the water medium:

-Si-O^−^ +H_2_O → -Si-O…H + OH^−^

-O-Si^+^ +H_2_O → -O-Si…OH + H^+^

Ca^2+^ + H_2_O → Ca(OH)^+^ + H^+^

Ca(OH)^+^ + H_2_O → Ca(OH)_2_ + H^+^

**Figure 9:**
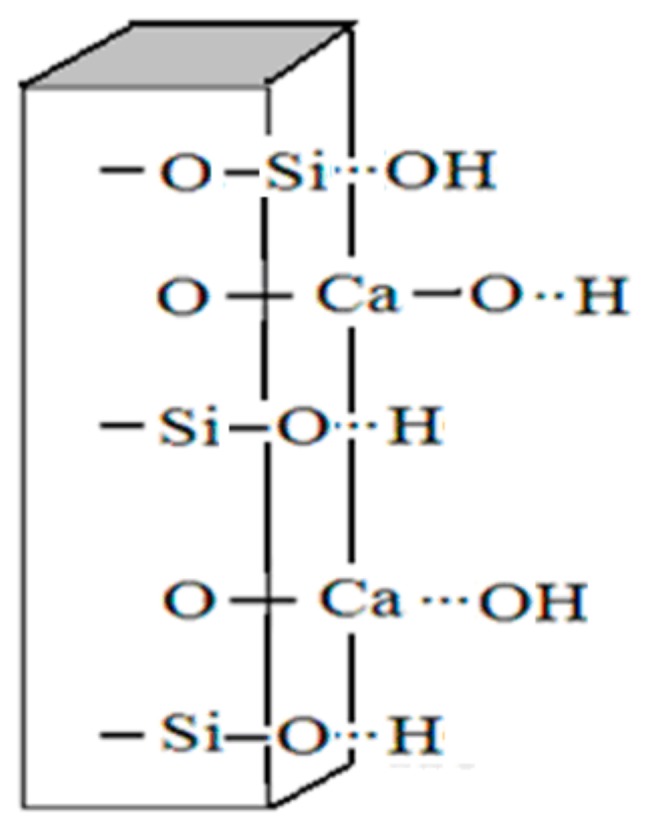
Surface components of wollastonite.

So, there are a certain number of active groups such as Si-OH and Ca-OH existing on the surface of wollastonite, which are the foundation for the dehydroxylation reactions with surface hydroxyl groups of TiO_2_. Figure 9 shows the surface functional groups of wollastonite in the water medium.

#### 3.3.2. The Surface Morphology of TiO_2_ Particles

**Figure 10:**
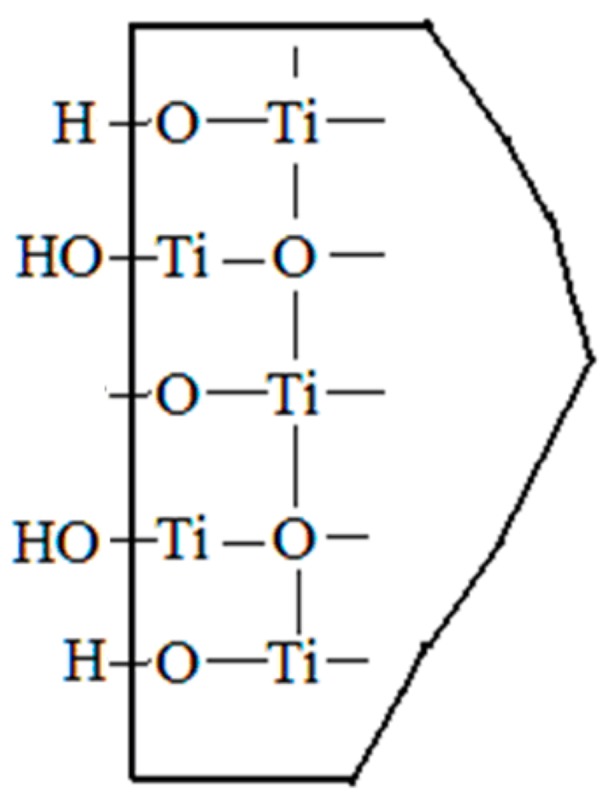
Hydroxylation on the surface of TiO_2._

TiO_2_ is one of the most typical oxides that can be hydroxylated on the surface, forming various types of hydroxyl groups on its surface. The surface’s unsaturated Ti^4+^ formed hydroxyl groups via different levels of hydration. According to the study by Bandara J [26], the anatase surface is electrically neutral at pH 4~9 and the surface’s unsaturated titanium (-Ti^+^) exists as -Ti OH, which indicates the hydroxylation on the surface of TiO_2_ particles, as shown in Figure 10.

#### 3.3.3. The Composite Model between Wollastonite and TiO_2_ Particles

**Figure 11:**
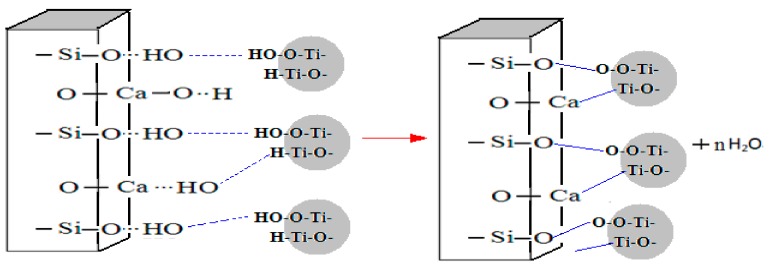
The interaction model on the surface of wollastonite and TiO_2_ particles.

According to the results and analysis above, the interaction model on the surface of wollastonite and TiO_2_ particles, in the process of preparing TiO_2_-coated wollastonite composite pigments by way of the mechano chemical method in a water medium, is established and shown in Figure 11.

## 4. Conclusions

(1) TiO_2_-coated wollastonite composite pigments were successfully prepared by way of the mechano-chemical method. The composite pigment (contains 45% TiO_2_) has similar oil absorption to titanium dioxide. The hiding power is 17.97 g/m^2^, reaching 81.08% of titanium dioxide with an increase of 36.08% compared to the same amount of TiO_2_ used in composite particles.
(2) A firm combination between wollastonite and TiO_2_ particles is formed through a dehydroxylation reaction, leading the composite materials to have the structure of TiO_2_ coating on the wollastonite surface evenly and closely. The wollastinite-TiO_2_ composite materials have similar properties to titanium dioxide.

## Figures and Tables

**Table 1:** The main properties of TiO_2_-coated wollastonite composite pigments and comparison.

Samples	Oil Absorption/(g/100 g)	Whiteness/%	Hiding Power/(g/m^2^)	Relative Hiding Power (*E*)/%	Δ*E*(*E* − *E*_0_)/%
TiO_2_-coated wollastonite composite pigments	22.72	96.6	17.97	81.08	36.08
Wollastonite and TiO_2_ dry mixtures	19.70	96.0	23.04	63.24	18.24
Wollastonite and TiO_2_ wet mixtures	20.12	96.3	21.56	67.58	22.58
Anatase TiO_2_	25.03	96.2	14.57	100	-
Wollastonite	11.20	94.0	272.65	-	-
